# Quantum Simulation of Dissipative Processes without Reservoir Engineering

**DOI:** 10.1038/srep09981

**Published:** 2015-05-29

**Authors:** R. Di Candia, J. S. Pedernales, A. del Campo, E. Solano, J. Casanova

**Affiliations:** 1 Department of Physical Chemistry, University of the Basque Country UPV/EHU, Apartado 644, 48080 Bilbao, Spain; 2 Department of Physics, University of Massachusetts, Boston, MA 02125, USA; 3 Theoretical Division, Los Alamos National Laboratory, Los Alamos, NM 87545, USA; 4 Center for Nonlinear Studies, Los Alamos National Laboratory, Los Alamos, NM 87545, USA; 5 IKERBASQUE, Basque Foundation for Science, Maria Diaz de Haro 3, 48013 Bilbao, Spain; 6 Institut für Theoretische Physik, Albert-Einstein-Allee 11, Universität Ulm, D-89069 Ulm, Germany

## Abstract

We present a quantum algorithm to simulate general finite dimensional Lindblad master equations without the requirement of engineering the system-environment interactions. The proposed method is able to simulate both Markovian and non-Markovian quantum dynamics. It consists in the quantum computation of the dissipative corrections to the unitary evolution of the system of interest, via the reconstruction of the response functions associated with the Lindblad operators. Our approach is equally applicable to dynamics generated by effectively non-Hermitian Hamiltonians. We confirm the quality of our method providing specific error bounds that quantify its accuracy.

While every physical system is indeed coupled to an environment^1^,^2^, modern quantum technologies have succeeded in isolating systems to an exquisite degree in a variety of platforms^3^,^4^,^5^,^6^. In this sense, the last decade has witnessed great advances in testing and controlling the quantum features of these systems, spurring the quest for the development of quantum simulators^7^,^8^,^9^,^10^. These efforts are guided by the early proposal of using a highly tunable quantum device to mimic the behavior of another quantum system of interest, being the latter complex enough to render its description by classical means intractable. By now, a series of proof-of-principle experiments have successfully demonstrated the basic tenets of quantum simulations revealing quantum technologies as trapped ions^11^, ultracold quantum gases^12^, and superconducting circuits^13^ as promising candidates to harbor quantum simulations beyond the computational capabilities of classical devices.

It was soon recognised that this endeavour should not be limited to simulating the dynamics of isolated complex quantum systems, but should more generally aim at the emulation of arbitrary physical processes, including the open quantum dynamics of a system coupled to an environment. Tailoring the complex nonequilibrium dynamics of an open system has the potential to uncover a plethora of technological and scientific applications. A remarkable instance results from the understanding of the role played by quantum effects in the open dynamics of photosynthetic processes in biological systems^14^,^15^, recently used in the design of artificial light-harvesting nanodevices^16^,^17^,^18^. At a more fundamental level, an open-dynamics quantum simulator would be invaluable to shed new light on core issues of foundations of physics, ranging from the quantum-to-classical transition and quantum measurement theory^19^ to the characterization of Markovian and non-Markovian systems^20^,^21^,^22^. Further motivation arises at the forefront of quantum technologies. As the available resources increase, the verification with classical computers of quantum annealing devices^23^^,^^24^, possibly operating with a hybrid quantum-classical performance, becomes a daunting task. The comparison between different experimental implementations of quantum simulators is required to establish a confidence level, as customary with other quantum technologies, e.g., in the use of atomic clocks for time-frequency standards. In addition, the knowledge and control of dissipative processes can be used as well as a resource for quantum state engineering^25^.

Facing the high dimensionality of the Hilbert space of the composite system made of a quantum device embedded in an environment, recent developments have been focused on the reduced dynamics of the system that emerges after tracing out the environmental degrees of freedom. The resulting nonunitary dynamics is governed by a dynamical map, or equivalently, by a master equation^1^,^2^. In this respect, theoretical^26^,^27^,^28^ and experimental^29^ efforts in the simulation of open quantum systems have exploited the combination of coherent quantum operations with controlled dissipation. Notwithstanding, the experimental complexity required to simulate an arbitrary open quantum dynamics is recognised to substantially surpass that needed in the case of closed systems, where a smaller number of generators suffices to design a general time-evolution. Thus, the quantum simulation of open systems remains a challenging task.

In this Letter, we propose a quantum algorithm to simulate finite dimensional Lindblad master equations, corresponding to Markovian or non-Markovian processes. Our protocol shows how to reconstruct, up to an arbitrary finite error, physical observables that evolve according to a dissipative dynamics, by evaluating multi-time correlation functions of its Lindblad operators. We show that the latter requires the implementation of the unitary part of the dynamics in a quantum simulator, without the necessity of physically engineering the system-environment interactions. Moreover, we demonstrate how these multi-time correlation functions can be computed with a reduced number of measurements. We further show that our method can be applied as well to the simulation of processes associated with non-Hermitian Hamiltonians. Finally, we provide specific error bounds to estimate the accuracy of our approach.

Consider a quantum system coupled to an environment whose dynamics is described by the von Neumann equation 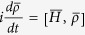. Here, 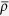 is the system-environment density matrix, 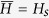

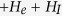
, where *H*_*s*_ and *H*_*e*_ are the system and environment Hamiltonians, while *H*_*I*_ corresponds to their interaction. Assuming weak coupling and short time-correlations between the system and the environment, after tracing out the environmental degrees of freedom we obtain the Markovian master equation


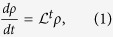


being 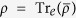 and 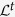 the time-dependent superoperator governing the dissipative dynamics^1^,^2^. Notice that there are different ways to recover Eq. (1)^30^. Nevertheless, Eq. (1) is our starting point, and in the following we show how to simulate this equation regardless of its derivation. Indeed, our algorithm does not need to control any of the approximations done to achieve this equation. We can decompose 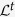 into 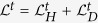. Here, 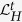 corresponds to a unitary part, i.e. 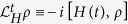, where *H*(*t*) is defined by *H*_*S*_ plus a term due to the lamb-shift effect and it may depend on time. Instead, 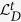 is the dissipative contribution and it follows the Lindblad form^31^ 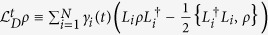, where *L*_*i*_ are the Lindblad operators modelling the effective interaction of the system with the bath that may depend on time, while 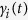 are nonnegative parameters. Notice that, although the standard derivation of Eq. (1) requires the Markov approximation, a non-Markovian equation can have the same form. Indeed, it is known that if 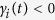 for some *t* and 
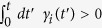
 for all *t*, then Eq. (1) corresponds to a completely positive non-Markovian channel^32^. Our approach can deal also with non-Markovian processes of this kind, keeping the same efficiency as the Markovian case. While we will consider the general case 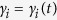, whose sign distinguishes the Markovian processes from the non-Markovian ones, for the sake of simplicity we will consider the case 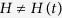 and 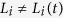 (in the following, we will denote 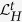 simply as 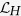). However, the inclusion in our formalism of time-dependent Hamiltonians and Lindblad operators is straightforward.

One can integrate Eq. (1) obtaining a Volterra equation^33^


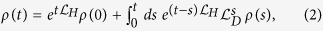


where 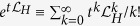. The first term at the right-hand-side of Eq. (2) corresponds to the unitary evolution of 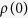 while the second term gives rise to the dissipative correction. Our goal is to find a perturbative expansion of Eq. (2) in the 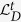 term, and to provide with a protocol to measure the resulting expression in a unitary way. In order to do so, we consider the iterated solution of Eq. (2) obtaining


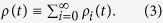


Here, 
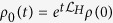
, while, for *i* ≥ 1, 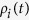 has the following general structure: 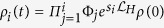 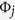 being a superoperator acting on an arbitrary matrix 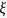 as 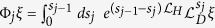, where 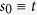. For instance, 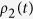 can be written as


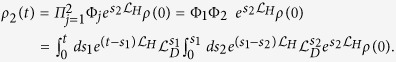


In this way, Eq. (3) provides us with a general and useful expression of the solution of Eq. (1). Let us consider the truncated series in Eq. (3), that is 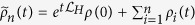, where *n* corresponds to the order of the approximation. We will prove that an expectation value 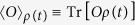 corresponding to a dissipative dynamics can be well approximated as


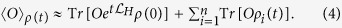


In the following, we will supply with a quantum algorithm based on single-shot random measurements to compute each of the terms appearing in Eq. (4), and we will derive specific upper-bounds quantifying the accuracy of our method. Notice that the first term at the right-hand-side of Eq. (4), i.e. 
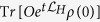
, corresponds to the expectation value of the operator *O* evolving under a unitary dynamics, thus it can be measured directly in a unitary quantum simulator where the dynamics associated with the Hamiltonian *H* is implementable. However, the successive terms of the considered series, i.e. 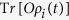 with *i* ≥ 1, require a specific development because they involve multi-time correlation functions of the Lindblad operators and the operator *O*.

Let us consider the first order term of the series in Eq. (4)


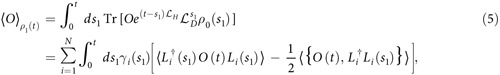


where 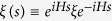 for a general operator 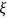 and time *s*, and all the expectation values are computed in the state 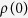. Note that the average values appearing in the second and third lines of Eq. (5) correspond to time correlation functions of the operators *O*, *L*_*i*_, 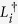, and 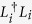. In the following, we consider a basis 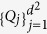, where *d* is the system dimension and *Q*_*j*_ are Pauli-kind operators, i.e. both unitary and Hermitian (see supplemental material^37^ for more details). The operators *L*_*i*_ and *O* can be decomposed as 
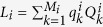
 and 
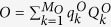
, with 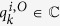, 
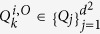
, and *M*_*i*_,*M*_*o*_ ≤ *d*^2^. We obtain then


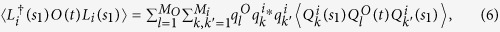


that is a sum of correlations of unitary operators. The same argument applies to the terms including 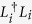 in Eq. (5). Accordingly, we have seen that the problem of estimating the first-order correction is moved to the measurement of some specific multi-time correlation functions involving the 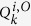 operators. The argument can be easily extended to higher-order corrections. Indeed, for the *n*-th order, we have to evaluate the quantity


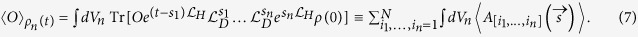


Here,


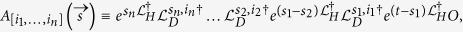


where 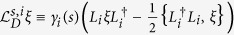, 
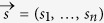
, 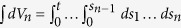, and 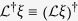 for a general superoperator 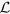. As in Eq. (5), the above expression contains multi-time correlation functions of the Lindblad operators 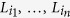 and the observable *O*, that have to be evaluated in order to compute each contribution in Eq. (4).

Our next step is to provide a method to evaluate general terms as the one appearing in Eq. (7). The standard approach to estimate this kind of quantities corresponds to measuring the expected value 
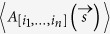
 at different random times 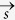 in the integration domain, and then calculating the average. Nevertheless, this strategy involves a huge number of measurements, as we need to estimate an expectation value at each chosen time. Our technique, instead, is based on single-shot random measurements and, as we will see below, it leads to an accurate estimate of Eq. (7). More specifically, we will prove that


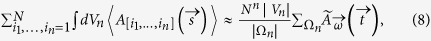


where 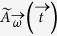 corresponds to a single-shot measurement of 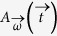, being 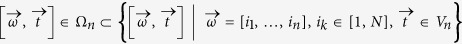, 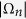 is the size of 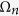, and 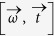 are sampled uniformly and independently. As already pointed out, the integrand in Eq. (7) involves multi-time correlation functions. In this respect, we note that a quantum algorithm for their efficient reconstruction has recently been proposed^34^. Indeed, the authors in Ref. [34] show how, by adding only one ancillary qubit to the simulated system, general time-correlation functions are accessible by implementing only unitary evolutions of the kind 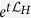, together with entangling operations between the ancillary qubit and the system. It is noteworthy to mention that these operations have already experimentally demonstrated in quantum systems as trapped ions^35^ or quantum optics^6^, and have been recently proposed for cQED architectures^36^. Moreover, the same quantum algorithm allows us to measure single-shots of the real and imaginary part of these quantities providing, therefore, a way to compute the term at the right-hand-side of Eq. (8). Notice that the evaluation of each term 
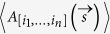
 in Eq. (7), requires a number of measurements that depends on the observable decomposition, see Eq. (6). After specifying it, we measure the real and the imaginary part of the corresponding correlation function. Finally, in the supplemental material^37^ we prove that


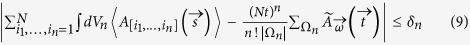


with probability higher than 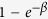, provided that 
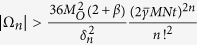
, where 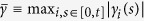 and 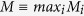. Equation (9) means that that the quantity in Eq. (7) can be estimated with arbitrary precision by random single-shot measurements of 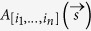, allowing, hence, to dramatically reduce the resources required by our quantum simulation algorithm. Notice that the required number of measurements to evaluate the order *n* is bounded by 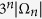, and the total number of measurements needed to compute the correction to the expected value of an observable up the order *K* is bounded by 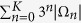. In the following, we discuss at which order we need to truncate in order to have a certain error in the final result.

So far, we have proved that we can compute, up to an arbitrary order in 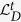, expectation values corresponding to dissipative dynamics with a unitary quantum simulation. It is noteworthy that our method does not require to physically engineer the system-environment interaction. Instead, one only needs to implement the system Hamiltonian *H*. In this way we are opening a new avenue for the quantum simulation of open quantum dynamics in situations where the complexity on the design of the dissipative terms excedes the capabilities of quantum platforms. This covers a wide range of physically relevant situations. One example corresponds to the case of fermionic theories where the encoding of the fermionic behavior in the degrees of freedom of the quantum simulator gives rise to highly delocalized operators^38^,^39^. In this case a reliable dissipative term should act on these non-local operators instead of on the individual qubits of the system. Our protocol solves this problem because it avoids the necessity of implementing the Lindblad superoperator. Moreover, the scheme allows one to simulate at one time a class of master equations corresponding to the same Lindblad operators, but with different choices of 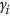, including the relevant case when only a part of the system is subjected to dissipation, i.e. 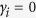 for some values of *i*.

We shall next quantify the quality of our method. In order to do so, we will find an error bound certifying how the truncated series in Eq. (3) is close to the solution of Eq. (1). This error bound will depend on the system parameters, i.e. the time *t* and the dissipative parameters 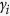. As figure of merit we choose the trace distance, defined by


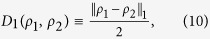


where 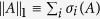, being 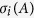 the singular values of *A*^40^. Our goal is to find a bound for 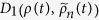, where 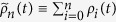 is the series of Eq. (3) truncated at the *n*-th order. We note that the the following recursive relation holds


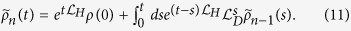


From Eq. (11), it follows that


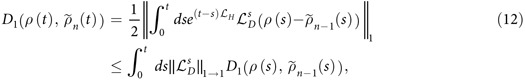


where we have introduced the induced superoperator norm 
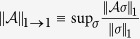
40. For *n* = 0, i.e. for 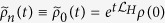, we obtain the following bound


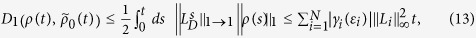


where 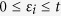
37, and 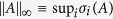. Notice that, in finite dimension, one can always renormalize 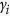 in order to have 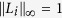, i.e. if we transform 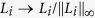, 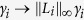, the master equation remains invariant. Using Eq. (12)–(13), one can show by induction that for the general *n*-th order the following bound holds


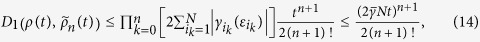


where 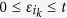 and we have set 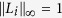. From Eq. (14), it is clear that the series converges uniformly to the solution of Eq. (1) for every finite value of *t* and choices of 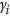. As a result, the number of measurements needed to simulate a certain dynamics at time *t* up to an error 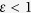 is *O* 
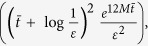
 where 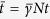
37. Here, a discussion on the efficiency of the method is needed. From the previous formula, we can say that our method performs well when *M* is low, i.e. in case where each Lindblad operators can be decomposed in few Pauli-kind operators. Moreover, as our approach is perturbative in the dissipative parameters 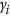, the method is more efficient when 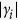 are small. Notice that analytical perturbative techniques are not available in this case, because the solution of the unperturbed part is assumed to be not known. Lastly, it is evident that the algorithm is efficient for certain choices of time, and the relevance of the simulation depends on the particular cases. For instance, a typical interesting situation is a strongly coupled Markovian system. Let us assume a site-independent couple parameter *g* and a dissipative parameter *γ*. We have that 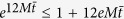 if 
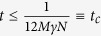
. In this period, the system oscillates typically 
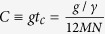
 times, so the simulation can be considered efficient for 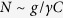, which, in the strong coupling regime, can be of the order of 10^3^/*C*. Notice that, in most relevant physical cases, the number of Lindblad operators *N* is of the order of the number of system parties^27^.

All in all, our method is aimed to simulate a different class of master equations with respect to the previous approaches, including non-Markovian quantum dynamics, and it is efficient in the range of times where the exponential 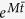 may be truncated at some low order. A similar result is achieved by the authors of Ref. [27], where they simulate a Lindblad equation via Trotter decomposition. They show that the Trotter error is exponentially large in time, but this exponential can be truncated at some low order by choosing the Trotter time step Δ*t* sufficiently small. Our method is qualitatively different, and it can be applied also to analogue quantum simulators where suitable entangled gates are available.

Lastly, we note that this method is also appliable to simulate dynamics under a non-Hermitian Hamiltonian 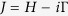, with 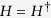, 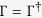. This type of generator emerges as an effective Hamiltonian in the Feshbach partitioning formalism^41^, when one looks for the evolution of the density matrix projected onto a subspace. The new Schrödinger equation reads


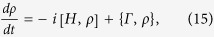


This kind of equation is useful in understanding several phenomena, e.g. scattering processes^42^ and dissipative dynamics^43^, or in the study of *PT*-symmetric Hamiltonian^44^. Our method consists in considering the non-Hermitian part as a perturbative term. As in the case previously discussed, similar bounds can be easily found (see the supplemental material^37^), and this proves that the method is reliable also in this situation.

In conclusion, we have proposed a method to compute expectation values of observables that evolve according to a generalized Lindblad master equation, requiring only the implementation of its unitary part. Through the quantum computation of *n*-time correlation functions of the Lindblad operators, we are able to reconstruct the corrections of the dissipative terms to the unitary quantum evolution without reservoir engineering techniques. We have provided a complete recipe that combines quantum resources and specific theoretical developments to compute these corrections, and error-bounds quantifying the accuracy of the proposal and defining the cases when the proposed method is efficient. Our technique can be also applied, with small changes, to the quantum simulation of non-Hermitian Hamiltonians. The presented method provides a general strategy to perform quantum simulations of open systems, Markovian or not, in a variety of quantum platforms.

## Additional Information

**How to cite this article**: Di Candia, R. *et al.* Quantum Simulation of Dissipative Processes without Reservoir Engineering. *Sci. Rep.* **5**, 9981; doi: 10.1038/srep09981 (2015).
